# A systematic review of the challenges, emerging solutions and applications, and future directions of PET/MRI in Parkinson’s disease

**DOI:** 10.1186/s41824-024-00194-9

**Published:** 2024-02-14

**Authors:** Isabella Hoi Kei Leung, Mark William Strudwick

**Affiliations:** 1 School of Health Sciences, Western Sydney University, Campbelltown, NSW Australia; 2 Centre for Advanced Imaging, University of Queensland, Brisbane, QLD Australia

**Keywords:** PET/MRI, Parkinson’s disease, Challenges, Applications, Translational research

## Abstract

PET/MRI is a hybrid imaging modality that boasts the simultaneous acquisition of high-resolution anatomical data and metabolic information. Having these exceptional capabilities, it is often implicated in clinical research for diagnosing and grading, as well as tracking disease progression and response to interventions. Despite this, its low level of clinical widespread use is questioned. This is especially the case with Parkinson’s disease (PD), the fastest progressively disabling and neurodegenerative cause of death. To optimise the clinical applicability of PET/MRI for diagnosing, differentiating, and tracking PD progression, the emerging novel uses, and current challenges must be identified. This systematic review aimed to present the specific challenges of PET/MRI use in PD. Further, this review aimed to highlight the possible resolution of these challenges, the emerging applications and future direction of PET/MRI use in PD. EBSCOHost (indexing CINAHL Plus, PsycINFO) Ovid (Medline, EMBASE) PubMed, Web of Science, and Scopus from 2006 (the year of first integrated PET/MRI hybrid system) to 30 September 2022 were used to search for relevant primary articles. A total of 933 studies were retrieved and following the screening procedure, 18 peer-reviewed articles were included in this review. This present study is of great clinical relevance and significance, as it informs the reasoning behind hindered widespread clinical use of PET/MRI for PD. Despite this, the emerging applications of image reconstruction developed by PET/MRI research data to the use of fully automated systems show promising and desirable utility. Furthermore, many of the current challenges and limitations can be resolved by using much larger-sampled and longitudinal studies. Meanwhile, the development of new fast-binding tracers that have specific affinity to PD pathological processes is warranted.

## Highlights

- The current PD diagnostic process is lengthy, with PD often being misdiagnosed as other parkinsonism disorders and other Lewy body disease.
- PET/MRI is a robust tool used primarily in oncology rather than neurological disorders such as PD.
- The key challenges include limited machinery, operational expertise, and feasibility concerns for late-severe PD stage.
- The clinical utility of PET/MRI in PD can be greatly enhanced via the use of fully automated systems and the development of short-lived radio-tracers such as [^11^-C] variations, and the use of faster binding tracers such as [^18^F]LBT-999.

## Background

Simultaneous positron emission tomography and magnetic resonance imaging (PET/MRI) is a non-invasive tool with robust clinical and research utility. This hybrid system integrates the high spatial resolution and soft tissue contrast capabilities of MRI, with the quantitative molecular detail capacity of PET. It was first successfully implemented in 2006 and undoubtedly is considered an exceptional imaging modality for diagnosing and grading, as well as tracking disease progression and response to interventions (Mannheim et al. 2018; Musafargani et al. 2018). Despite this, it remains a novel and emerging technology that is only slowly entering widespread use within the clinical environment (Vitor et al. 2017b). By contrast, PET/MRI is quickly becoming the modality of choice in the field of neuroscience, translational neurological/psychiatric, and drug development research (Catana et al. 2012). Within an ageing population context, there is a particular urgency for more advanced tools and techniques which aims to achieve earlier detection, enhanced efficiency, and accuracy of diagnosis for ageing disorders such as those leading to neurodegeneration. This is to enable the development of targeted interventions to delay the progression and combat the growing prevalence of expensive and burdensome neurodegenerative disorders. Specifically, the World Health Organisation (WHO) has reported that disability and death caused by Parkinson’s disease (PD) are increasing faster than for any other neurological disorder (World Health Organisation 2022a).

PD is the second most common neurodegenerative disorder following Alzheimer’s disease and is the leading cause of “parkinsonism” (Parkinson’s Australia 2022). Parkinsonism refers to motor disturbances including rigidity, postural instability, bradykinesia, and tremor (Tolosa et al. 2006). Additionally, cognitive impairment, mental health conditions, pain, and other sensory dysfunction may manifest (World Health Organisation 2022a). Unfortunately, the process of PD diagnosis remains lengthy, and PD is commonly misdiagnosed as other parkinsonism disorders such as multiple system atrophy (MSA), vascular parkinsonism, or progressive supranuclear palsy among others (Schrag et al. 2002; Beach and Adler 2018). Further, although there are pathological hallmarks of PD (dopaminergic neuronal dysfunction in the substantia nigra pars compacta and Lewy-body formation from misfolded α-synuclein proteins) (MacMahon Copas et al. 2021; Dickson 2018; Levin et al. 2016), it is difficult to distinguish PD from other Lewy body disease such as Lewy-body dementia (LBD), idiopathic rapid eye movement sleep behaviour disorder, and pure autonomic failure, especially during the early disease stages (Levin et al. 2016; Sezgin et al. 2019). The commonality of misdiagnosis may be attributable to the fact that (World Health Organisation 2022b). Hence, utilising more advanced tools and techniques to enhance efficiency and accuracy of diagnostic methods are required to facilitate early and targeted intervention, thereby delaying the progression and reducing socioeconomic burden.

The use of PET/MRI hybrid systems compared to each individual modalities in PD is appreciated to be compelling with respect to providing simultaneous metabolic, structural, and functional insight (Shang et al. 2021a; Ruppert et al. 2020). For example, PET/MRI has detected early asymmetric pattern of dopaminergic dysfunction in the putamen and disruption of nigrostriatal pathway integrity in PD compared to healthy-matched controls (Shang et al. 2021a). Further, these microstructural changes were significantly associated with poorer motor performance which was mediated by putaminal molecular degeneration (Shang et al. 2021a). In addition to early detection of pathological changes, PET/MRI has also been used to distinguish PD from other parkinsonian disorders and those with Lewy-body pathology. Of note, in concordance with previous PET studies (Brajkovic et al. 2017; Meyer et al. 2017; Eckert et al. 2005), Hu and colleagues found that there are normal or slightly elevated levels of fluorodeoxyglucose (a measure of glucose consumption) ([^18^F]-FDG) uptake in the striata of early PD, compared to MSA which conversely displays hypometabolism in the putamen, pons, and cerebellum (Hu et al. 2021a). On the other hand, whilst PD and LBD appear similar using [^18^F]-FDG PET/MRI (Vitor et al. 2017b), parkinsonian syndromes can be better identified using dopaminergic synthesis and transmission tracers such as the dopamine precursor tracer 3,4-dihydroxy-6-[^18^F]-fluoro-1-phenylalanine ([^18^F] FDOPA) or the presynaptic transporter-targeting [^18^F] fluoropropyl-carbomethoxyiodiohenylnortropane ([^18^F]FP-CIT) (Barthel et al. 2015). Regardless, the synergistic advantage of PET/MRI over its individual components is exemplified through the finding that concurrent acquisition of metabolic, structural, and functional data significantly improves efficiency, and minimises inaccurate spatial alignment, and segmentation errors in regions of interest (ROI) (Hu et al. 2021a). This is important as sensitivity to metabolic changes at microscopic levels being aligned with precise anatomical features, can augment the characterisation of PD in relation to other parkinsonian and Lewy-body disorders.

Provided that PET/MRI is seemingly a promising robust solution in enhancing the efficiency and accuracy of PD diagnosis, the challenges and limitations that prevent its widespread translational clinical utility are unclear. It was therefore the primary aim of this systematic review to identify the key challenges of PET/MRI use in PD. Further, this review highlighted the possible resolution of these challenges, the emerging applications, and future direction of PET/MRI use in PD for clinical settings.

## Methods

The conduct and reporting of this systematic review adhered to the Preferred reporting Items for Systematic Reviews and Meta-Analyses (PRISMA) 2020 Statement guidelines (Page et al. 2021).

This review is registered with PROSPERO International prospective register of systematic reviews: CRD42022383414.

### Eligibility criteria

A search was conducted for peer-reviewed literature published in EBSCOHost (indexing CINAHL Plus, PsycINFO) Ovid (Medline, EMBASE) PubMed, Web of Science, and Scopus from 2006 (the year of first integrated PET/MRI hybrid system) to 30 September 2022. The population, intervention, comparison, outcome, study design (PICOS) framework was used to develop the review selection criteria.

Articles eligible for inclusion:

- Published in English
- Population: Parkinson’s disease
- Intervention: PET/MRI hybrid systems
- Comparisons: routine imaging protocols, including independent PET or MRI, or the use of other hybrid systems such as PET/CT, routine data-processing tools, healthy controls, other neurodegenerative diseases
- Outcomes: applications, challenges/limitations, future directions
- Study design: primary studies (randomised controlled trials, cohort studies, case–control studies, cross-sectional studies, case reports, and case studies)

Studies were excluded if: a full text could not be obtained; non-human studies; study protocols; published in a format other than a journal article (book chapter, thesis, editorial, letter, webpage etc.); secondary studies (reviews, essays, commentaries, opinions); studies where the data from PD patients could not be extracted (general neurological disorders, neurodegenerative disease, motor disorders); studies where imaging was not conducted on PET/MRI hybrid systems.

### Search strategy and study selection

Due to the qualitative nature of the outcomes for this review, a broad search strategy was conducted. The search terms consisted of all potential synonyms and combinations of “Parkinson’s disease” and “PET/MRI” (MeSH terms were selected where applicable). The following search was completed in Ovid (indexing Medline and EMBASE) with restricted year range between 2006 and 2022 (Table 1).

**Table 1:** Search strategy used to identify articles in Ovid search engine

Number	Search items
#1	[All fields] Parkinson’s disease.mp or Parkinson Disease/
#2	[All fields] PET-MRI.mp
#3	[All fields] Positron emission tomography/ and magnetic resonance imaging
#4	#2 OR #3
#5	#1 AND #4

**Fig. 1:**
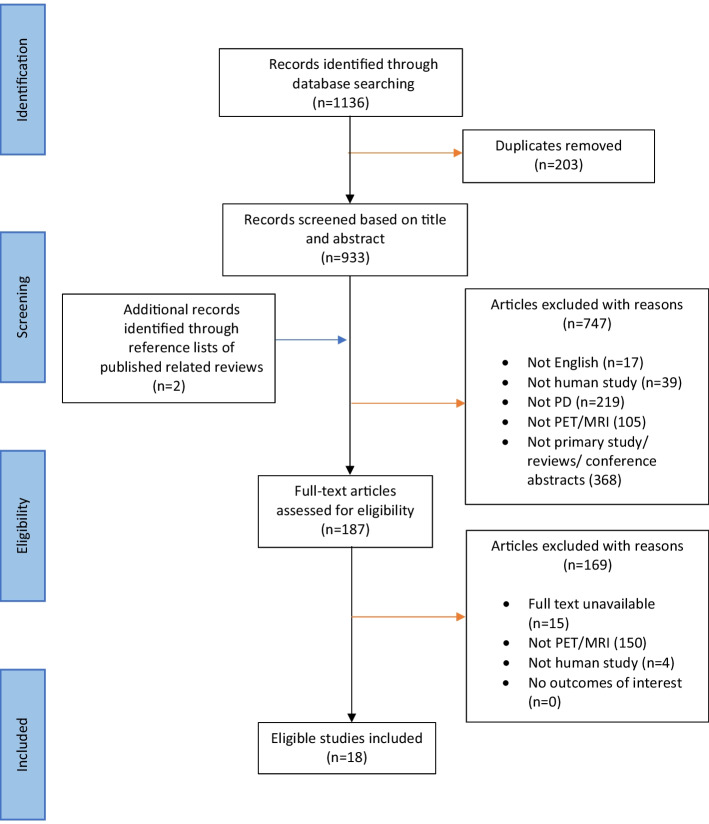
Flowchart of article identification and screening

The references of retrieved articles were then imported into Endnote (version X9) for further screening. Following the removal of duplicates, the titles and abstracts were screened based on the selection criteria and potentially eligible articles were grouped for full-text evaluation**.** The reference list of related published reviews and primary studies was also screened for potential articles to be added to this review. Further full-text screening was then conducted based on the outcomes of this study. Those that reported or discussed emerging applications, challenges/limitations, and future directions of PET/MRI in PD were included in this review (Fig. 1).

### Data extraction and synthesis

Study characteristics were extracted from included studies. The following data was extracted from each of the studies: first author, year of publication, location, study population (including disease staging), number of participants (intervention and controls), age (mean, SD), PET tracer used with MRI sequences conducted, and summary of findings.

## Results

### Study selection

Figure 1 represents the study selection process for this review and the number of studies identified at each stage. A total of 1136 records were identified with 933 remaining after the removal of duplicates. Eighteen articles met eligibility criteria for this systematic review.

### Study characteristics

The characteristics of the 18 included studies are shown in Table 2. Studies were conducted between years 2014 and 2022. There were several studies that used overlapping patient samples (two studies from Korea An et al. 2016; Choi et al. 2016 used overlapping samples, and two studies from Italy Biundo et al. 2021; Garon et al. 2022 used overlapping samples) giving a total of fifteen unique study cohorts. The majority of studies were conducted in China (five studies) followed by Korea (four studies) and Italy (three studies). The following countries had one study each: Norway, USA, Belgium, India, Spain, Germany. Of the 18 studies, nine studies included PD in the early stages (Hoehn & Yahr score1-3 mild-moderate severity) (Hoehn and Yahr 1998) one study (Zang et al. 2022) included individuals with moderate–severe PD (Hoehn & Yahr score 4–5 indicates severe) (Hoehn and Yahr 1998). Eleven studies reported the Unified Parkinson’s Disease Rating Scale UPDRS III (Item III representing motor dysfunction severity) and/or UPDRS total scores (0 indicating absence of signs and symptoms, maximum score of 199 in UPDRS indicating the most severe level of disability due to PD). The UPDRS total score is a summed measurement score including subscale I for mentation, behaviour and mood, subscale II for activities of daily living, and subscale III for motor examination (Ramaker et al. 2002). Six studies did not report on either Hoehn & Yahr stage or UPDRS scales.

**Table 2:** Study Characteristics of the eighteen identified PET/MRI hybrid studies in patients with Parkinson’s disease

Lead author (year); location	Population	Number of participants	Mean age ± SD years	PET tracer/ MR protocol	Summary of findings
An et al. (2016) Korea*	PD UPDRS III score 7.6 ± 4.5 Hoehn & Yahr score 1.4 ± 0.5 HC Healthy normal volunteers	PD (*n* = 20) HC (*n* = 10)	58.7 ± 7.3	[^18^F]-FP-CIT Images acquired 110 min after injection [^18^F]-FDG Images acquired 40 min after injection Dual-echo UTE sequence (TE 0.07 and 2.46 ms, TR = 11.9 ms, flip angle = 10°, slice = 1.6, matrix = 192 × 192 × 192 with isotropic voxel size = 1.33 mm, FOV = 300 × 300 mm) T1-weighted 3D ultrafast GE sequence matrix = 208 × 256 × 256 with voxel size = 1.0 × 0.98 × 0.98 mm)	The MR-based attenuation correction including bones using UTE using level-set segmentation with inhomogeneity correction was demonstrated to be a feasible method with the tracers used The attenuation maps generated by MR using level-set segmentation and PET images were superior compared to corrected attenuation and scatter offered by the manufacturer of the PET/MRI
Biundo et al. (2021) Italyϕ	PD with normal cognition UPDRS total score 60.1 ± 33.5 UPDRS III score 36.3 ± 21.5 MCI Dementia LBD	PD (*n* = 5) MCI (*n* = 22) Dementia (*n* = 13) LBD (*n* = 10)	71.5 ± 5.7	[^18^F]-flutemetamol Images acquired 0–10 and 90–110 min after injection UTE used for attenuation correction T1-weighted 3D MPRAGE (TE = 2.53 ms, TR = 1.9 ms, slice = 1 mm, matrix = 256 × 256, FOV = 250) 1 mm isotropic T2-weighted 3D, and 2D SWI were acquired for clinical evaluation to exclude secondary parkinsonisms, vascular brain damage and to allow visual rating scales assessment	The presence of amyloid beta does not differentiate the clinical profile between PD and LBD
Brakedal et al. (2022) Norway	Newly diagnosed, drug-naïve PD (treatment with nicotinamide riboside versus placebo) UPDRS total score 43 ± 13.3 UPDRS III score 29 ± 9.35	PD (treatment with nicotinamide riboside) (*n* = 15) PD (placebo) (*n* = 15)	63.8 ± 9.5	[^18^F]-FDG Imaging acquired 25–30 min after injection T1-weighted 3D MPRAGE (TE = 2.26 ms, TR = 2.4 s, TI = 900 ms, flip angle = 8°, matrix = 256 × 256 × 192, FOV = 256 × 256 × 192) MRS ^31^P spectroscopy 3D chemical shift imaging FID sequence For calculating field-strength dependent chemical shift difference, relative amplitudes and frequency separations of oxidised (NAD +) and reduced NAD (NADH)	NR may be a promising neuroprotective medication for PD. Confirmation in longitudinal studies is required to determine the effects of NR on cerebral metabolism and cellular nicotinamide adenine dinucleotide (NAD) levels
Chen et al. (2019) USA	PD Unspecified clinical features and physical examinations AD MCI LBD HC	PD (*n* = 12) AD (*n* = 6) MCI (*n* = 2) LBD (*n* = 1) HC (*n* = 11)	67.7 ± 7.9	[^18^F]-florbetaben Imaging acquired 90–110 min after injection T1-weighted spoiled gradient recalled acquisition (TE = 3.092 ms, TR = 7.648 ms, TI = 400 ms, flip angle = 11°) T2-weighted FSE (TE = 100.896 ms, TR = 3484 ms, flip angle 111°) T2-FLAIR (TE = 161.488 ms, TR = 6000 ms, TI = 1773 ms, flip angle = 90°)	High-quality amyloid PET images can be generated by using deep-learning methods with ultra-low-dose PET data
Choi et al. (2014) Korea*	PD Unspecified clinical features and physical examinations Non-parkinsonian tremor	PD (*n* = 14) Non-parkinsonian tremor (*n* = 2)	61.3 ± 9.4	[^18^F]-FP-CIT Images acquired 110 min after injection Dual-echo UTE sequence (TE 0.07 and 2.46 ms, TR = 11.9 ms, flip angle = 10°, slice = 1.6, matrix = 192 × 192 × 192 with isotropic voxel size = 1.33 mm, FOV = 300 × 300 mm)	UTE sequence-based attenuation correction caused spatial bias despite attenuation maps accounting for cortical bones The DAT BR in the caudate nucleus was considerably underestimated due to the CSF space being misclassified as air in the lateral ventricles
Choi et al. (2016) Korea*	PD UPDRS III score 7.6 ± 4.5 Hoehn & Yahr score 1.4 ± 0.5 Non-parkinsonian tremor	PD (*n* = 16) Non-parkinsonian tremor (*n* = 7)	60.8 ± 9.5	[^18^F]-FP-CIT Images acquired 110 min after injection Dual-echo UTE sequence (TE 0.07 and 2.46 ms, TR = 11.9 ms, flip angle = 10°, slice = 1.6, matrix = 192 × 192 × 192 with isotropic voxel size = 1.33 mm, FOV = 300 × 300 mm) T1-weighted 3D MPRAGE (TE = 1.89 ms, TR = 1.67 ms, flip angle = 9°, matrix = 256 × 256, isotropic voxel = 1.0 mm, FOV = 250)	There is a close relationship between global structural changes and the degeneration of striatal dopaminergic circuits Although not statistically significant, there is a trend of positive correlation between anterior striatal dopaminergic degeneration with changes in the cerebellum and parahippocampal gyri. These may be associated with the non-motor circuits of the basal ganglia There was also a trend with the posterior striatal dopaminergic degeneration being negatively associated with grey matter density in the temporal and occipital cortex
Delva et al. (2020); Belgium	PD UPDRS total 41.5 ± 13.7 UPDRS III (off medication) 24.0 ± 7.2 Hoehn & Yahr score (On medication) 1 ± 1 (Off medication) 2 ± 0 HC age- and gender- matched	PD (*n* = 30) HC (*n* = 20)	60 ± 8.7	[^18^F]-FE-PE2I Images acquired 50–70 min after injection Performed on medication because antiparkinsonian drugs are considered not to interfere significantly with DAT imaging MR protocol not reported	In patients with early stage PD, the axon terminals appear to be the most vulnerable part of nigrostriatal dopaminergic neurons
Garon et al. (2022) Italyϕ	PD-MCI UPDRS total 49.7 ± 11.5 UPDRS III 28.4 ± 15.5 Hoehn & Yahr score 2.5 ± 1	PD-MCI Amyloid beta-positive (*n* = 8) PD-MCI Amyloid beta-negative (*n* = 17)	70.8 ± 5.5	[^18^F]-FMM Images were acquired between 0– 10 and 90–110 min after injection T1-weighted 3D MPRAGE (TE = 2.53 ms, TR = 1.9 ms, slice = 1 mm, matrix = 256 × 256, FOV = 250) 1 mm isotropic T2-weighted 3D, and 2D SWI were acquired for clinical evaluation	Amyloid burden in the fronto-striatal network may be associated in the worsening of executive functions in PD-MCI Within this cohort, amyloid accumulation was not related to cognitive deficits and brain atrophic patterns that are typically found in AD nor with specific clinical features (disease duration, age of motor symptom onset, medication dosage) or demographics
Hu et al. (2021) China	PD Unspecified stage and disease severity MSA	PD (*n* = 60) MSA (*n* = 30)	56 ± 9.2	[^18^F]-FDG Patients fasted for 6 h and stopped taking any medication for at least 12 h Image was acquired 1 h post injection T1-weighted (TE = 3.0 ms, TR = 7.9 ms, flip angle = 12°, matrix = 288 × 224, slice = 1 mm) T2-weighted (TE = 105 ms, TR = 5523 ms, refocusing flip angle = 142°, matrix = 384 × 240, slice = 5 mm, slice gap = 1.5 mm) T2-FLAIR (TE = 100 ms, TR = 9000 ms, TI = 2475 ms, refocusing flip angle = 160°, matrix = 256 × 192, slice = 5 mm, slice gap = 1.5 mm) fMRI SWI (TE = 4.0 ms, TR = 45.5 ms, flip angle = 15°, matrix = 384 × 320, slice = 3 mm) DWI (TE = 70.0 ms, TR = 6379 ms, matrix = 128 × 128, slice = 5 mm, slice gap = 1.5 mm)	The clinical-radiomics integrated model based on PET/MRI displayed higher performance (more accurate) compared to construction of radiomics signatures by the least absolute shrinkage and selection operator (LASSO) method to identify PD and MSA
Kwon et al. (2016) Korea	Suspected PD as diagnosed by experienced neurologist	Suspected PD (*n* = 15)	65.1 ± 10.4	[^18^F]-FP-CIT Images acquired 110 min after injection Antiparkinsonian drugs were stopped 12 h prior to the scans Dual UTE sequence (TE 0.07 and 2.46 ms, TR = 11.94 ms, flip angle = 10°, matrix = 192 × 192, FOV = 300 × 300 mm)	PET/MRI was found to be comparable to PET/CT in discriminating parkinsonian disorders
Mangalore et al. (2022) India	PD Disease status not reported FLD AD LBD PSP Corticobasal degeneration MND NPH	PD (*n* = 2) FLD (*n* = 16) AD (*n* = 9) LBD (*n* = 4) PSP (*n* = 1) Corticobasal degeneration (*n* = 2) MND (*n* = 1) NPH (*n* = 2)	Not reported	PET tracer and parameters not specified T1-weighted MPRAGE (TE = 2.4 ms, TR = 2200 ms, slice = 1 mm, FOV = 220 × 220 mm	This was bilateral frontal, parietal, and temporal atrophy (as with PSP) Patterns of atrophy correlated with areas of hypometabolism shown on PET
Pan et al. (2022) China	PD UPDRS III 21.5 ± 11.8 Hoehn & Yahr score 1.9 ± 0.9 HC	PD (*n* = 30 HC (*n* = 38)	56.3 ± 13.1	[^18^F]-FP-DTBZ Participants were scanned during their off-state condition (12 h after last medication) T1-weighted 3D imaging (TE 3.2 ms, TR = 7.86 ms, flip angle = 10°, voxel size = 0.5 × 0.5 × 0.67 mm, FOV = 230 × 230 mm)	Multi-atlas-based PET/MRI image segmentation method was superior than the template-based method. This method has potential value for enhancing the efficiency and accuracy of routine clinical PD diagnosis
Quarantelli et al. (2022) Italy	PD UPDRS III 20.4 ± 12.6 Hoehn & Yahr score 1.6 ± 0.6 ET	PD (*n* = 39) ET (*n* = 16)	68.6 ± 8.1	[^18^F]-DOPA Participants fasted for 4 h and discontinued antiparkinsonian medication for at least 12 h prior to scanning. 200 mg of carbidopa was administered 1 h before [^18^F]-DOPA injection to ensure maximal availability of DOPA to the brain and reduce bladder and kidney absorption Imaging was done 86.1 ± 21.8 min following the injection T2*-weighted single shot EPI (TE = 30 ms, TR = 2040 ms, 37 axial slices with 0.5 mm gap, flip angle = 90°, voxel = 3.5 × 3.5 × 3.0 mm, matrix = 64 × 64 mm) MPRAGE (TE = 2.3 ms, TR = 2300 ms, TI = 900 ms, 176 contiguous sagittal slices, flip angle = 8°, matrix = 256 × 248, voxel = 1 × 1 × 1 mm)	The functional connectivity pattern of the cortico-striatothalamic-cortical loop is influenced by nigrostriatal innervation in PD Decreasing metabolic values directly correlate with the connectivity of the sensorimotor cortex with the paracentral lobule and superior temporal regions. The clinical scores demonstrate that these in-turn could potentially cause loss of motor function There is an inverse correlation between the functional connectivity of the thalamus with the sensorimotor cortices that may represent ineffective compensatory mechanisms
Rodriguez-Rojas et al. (2020) Spain	PD UPDRS III 33.0 ± 6.4 Hoehn & Yahr score 1.6 ± 0.6	PD (*n* = 8)	61.1 ± 10.3	[^18^F]-FDG PET acquisition was done 40 min after injection Data was acquired with patients off-medication T1-weighted MPRAGE (TE = 3.34 ms, TR = 2300 ms, slice = 1 mm, matrix = 256 × 256, FOV = 256 × 256) SWI Parameters not specified T2-weighted TSE Parameters not specified	There were significant positive changes in glucose metabolism in distributed nodes o the cortical-subcortical networks following a incisionless transcranial intervention (MRgFUS-subthalamotomy). The procedure shifted metabolic patterns towards healthy values
Shang et al. (2021) China	Early stage PD UPDRS III 26.88.0 ± 8.73 Hoehn & Yahr score 1.28 ± 0.45 HC matched for age, sex, education, and Montreal Cognitive Assessment (MoCA) scores	Early stage PD (*n* = 25) HC (24)	65 ± 12.6	[^18^F]-DOPA Patients were fasted for at least 6 h. Carbidopa was orally administered 1 h prior to injection of [^18^F]-DOPA Data acquisition was done 90 min following injection Transverse EPI sequence-based DTI (TE = 78 ms, TR = 4663 ms, flip angle = 90°, slice = 4 mm, matrix = 118 × 128 mm, FOV = 230 × 250 mm, acceleration factor = 2 T1-weighed spoiled GRE (TE = 3.2 ms, TR = 7.86 ms, TI = 810 ms, slice = 0.71 mm, flip angle = 10°, matrix = 348 × 384 mm, FOV = 232 × 256 mm)	PET and DTI abnormalities and asymmetry of the dopaminergic system were detected in the early stage PD patients On the more affected side, there were significant associations between DTI metrics (nigral FA, putaminal MD and FA of the nigrostriatal projection) and motor performance. These were also significantly medicated putaminal standardised uptake value
Wimalarathne et al. (2022) China	Patients suspected of AD/PD	Patients suspected of AD/PD (*n* = 75)	61.2 ± 8.9	[^11^ C]-CFT Unspecified PET protocol [^11^ C]-PiB Unspecified PET protocol T1-weighted 3D GE sequence (TE 2.6 ms, TR = 6.9 ms, flip angle = 12°, matrix = 384 × 384, FOV = 240 × 240 mm)	Standard uptake value and image quality parameters were improved by time-of-flight reconstruction
Wurster et al. (2022) Germany	Rare SNCA-Triplication Early onset PD	Early onset PD (*n* = 1)	24	[^18^F]-FDG PET data was acquired 1 h after injection MPRAGE (TE = 3.37 ms, TR = 2300 ms, TI = 900 ms, voxel = 1.0 × 1.0 × 1.0 mm)	Bilateral hypometabolism in the frontal, temporoparietal, occipital, precuneus, gyrus cinguli posterior regions were noted in this patient. Corresponding left-sided atrophy was noted in these regions. Conversely, there was increase glucose metabolism in the basal ganglia with normal volumetric appearance
Zang et al. (2022) China	PD UPDRS III 59.71 ± 15.00 Hoehn & Yahr score 3.00 ± 0.83 HC age- and sex-matched	PD (*n* = 34) HC (*n* = 25)	61.2 ± 5.5	[^18^F]-FDG Patients fasted at least 6 h and did not take medication 12 h before examination Images were acquired on an average of 1 h after injection Resting-state fMRI SWI (TE = 30 ms, TR = 2000 ms, flip angle = 90°, slice = 3.5 mm, slice gap = 0.7 mm voxel = 3.5 × 3.5 × 3.5 mm, FOV = 230 × 230 mm High-resolution T1-weighted 3D imaging (TE = 3.8, TR = 7.9, FOV = 256 × 256 mm, Voxel = 1 × 1 × 1mm) 3D multi echo GE SWI (TE = 3.1/6.4/9.7/13.0/16.3/19.6 ms, TR = 29 ms, flip angle = 15°, matrix = 256 × 256 mm, voxel = 1 × 1 × 2mmacceleration factor = 2)	In PD patients, there was increased iron deposition in the substantia nigra, increased FDG uptake in the putamen bilaterally, and decrease in functional connectivity between the substantia nigra and the anterior putamen bilaterally There was also significant interaction between nigral iron deposition and nigral-putamen connectivity with putaminal FDG uptake

*PD* Parkinson’s disease, *HC* healthy control, *AD* Alzheimer’s disease, *MCI* mild cognitive impairment, *LBD* Lewy body dementia, *MSA* multiple system atrophy; *FLD* frontotemporal lobar degeneration, *PSP* progressive supranuclear palsy, *MND* motor-neuron disease, *NPH* normal pressure hydrocephalus, *ET* essential tremor, *TE* echo time, *TR* repetition time, *TI* inversion time, *GE* gradient echo, *FSE* fast spin echo, *FOV* field of view, *MPRAGE* magnetization prepared rapid acquisition gradient echo, *fMRI* functional MRI, *FLAIR* fluid attenuated inversion recovery, *SWI* susceptibility-weighted imaging, *DWI* diffusion weighted imaging, *EPI* echo planar imaging, *DTI* diffusion tensor imaging, *FA* fractional anisotropy, *MD* mean diffusivity; *[*^*18*^*F]-FP-CIT* [^18^F]-fluoropropyl-carbomethoxyiodophenylnortropane (a marker for dopaminergic nerve terminals to assess dopamine transporter binding), [^*18*^*F]-FDG* [^18^F]-Fluorodeoxyglucose (radiolabelled glucose molecule), *[*^*18*^*F]-FE-PE2I* [^18^F]-(E)-N-(3-iodoprop-2-enyl)-2β-carbofluoroethoxy-3β-(40-methyl-phenyl) nortropane (a selective dopamine transporter [DAT] radioligand to measure presynaptic and somatodendritic DAT levels of dopaminergic neurons), *[*^*18*^*F]-FMM* [^18^F]-flutemetamol (a robust and reliable marker for the detection of brain neuritic amyloid beta plaques), *[*^*18*^*F]-FP-DTBZ* [^18^F]-9-fluoropropyl-(+)-dihydrotetrabenazine (a marker for vesicular monoamine transporter type 2 [VMAT2] which is a transporter responsible for the uptake and storage of monoamines and an indicator of dopaminergic neuron integrity), *[*^*18*^*F]-DOPA* 3,4‑dihydroxy‑6‑[^18^F]-fluoro-l‑phenylalanine (a measure for the uptake of dopamine precursors to assess presynaptic dopaminergic integrity), *UTE* ultrashort echo time, *UPDRS III* unified Parkinson’s Disease Rating Scale (scale including Tremor, rigidity, bradykinesia), *UPDRS total* unified Parkinson’s Disease Rating Scale total score (scale including scale I for mentation, behaviour and mood, scale II for activities of daily living, and scale III for motor examination), *DAT BR* binding ratio of dopamine transporter, *CSF* cerebrospinal fluid, *[*^*11*^*C]-CFT* 11C-2-ß-carbomethoxy-3-b-(4-fluorophenyl) tropane (DAT tracer targeted for early PD diagnosis), *[*^*11*^*C]-PiB* 2-(4-N-[11C] methylaminophenyl)-6-hydroxybenzothiazole (used to image amyloid beta deposition), *MRgFUS* magnetic resonance-guided focused ultrasound

^*^Overlapping patient sample from Korea

ϕ Overlapping patient sample from Italy

The most commonly used PET tracer used was [^18^F]-FDG (6 studies) followed by the use of [^18^F]-FP-CIT (4 studies) and [^18^F]-DOPA (2 studies). Structural MRI mostly utilised T1-weighed MPRAGE sequences (*n* = 6) whilst functional MRI data was acquired using fMRI SWI by two articles (Hu et al. 2021b; Zang et al. 2022). Six studies indicated that PD patients were “off-medication”. On the other hand, one study reported that PD patients were “on-medication” during the PET/MRI examination (Delva et al. 2020).

### Quality of the studies

Due to the nature of the included studies (many of which are not RCTs, not blinded, and not intervention studies per se), the study quality and risk of bias was not assessed.

### Applications

Three studies discussed the translational clinical application of the PET/MR imaging and data-processing techniques. In the study by Wimalarathne and colleagues, it was noted that there is an advantage for using time-of-flight (TOF) reconstruction in PET/MR with short-lived [^11^C]-tracers as it offers higher sensitivity (Wimalarathne et al. 2022). The use of [^11^C]-tracers also has the advantage of shorter scan time, thereby improving patient comfort and reducing potential motion artefacts (that are common in patients with PD). There is, however, the challenge of combined TOF algorithm used as the authors found that it has differing effects on standard uptake values, contrast, and signal-to-noise ratio (SNR) of different brain regions (Wimalarathne et al. 2022).

Meanwhile to present another emerging application, the study by Chen and team proposed a reconstruction method that is able to reduce image noise from low-quality or low-dose PET, namely the use of convolutional neural networks. In addition, these authors found that multi-contrast MR inputs have been shown to recover the uptake patterns of the low-dose tracer. The results from the Chen study show that there is the potential for an increase in this form of low-dose amyloid scanning in clinical practice to inform PD and AD diagnostic workflows and track amyloid-targeting pharmaceuticals in future studies (Chen et al. 2019).

Finally, a fully automated multi-atlas database for PET image analysis developed by Pan et al. recently has shown great potential for PD diagnosis that is comparable and even superior (greater efficiency and accuracy) to conventional PET image-analysis methods such as manual, MR-based, MR-free-template-based methods (Pan et al. 2022).

### Challenges

Seven articles reported that small sample size was a major limitation of their respective studies. The low number of patients in these studies significantly impacts the statistical power and requires careful interpretation of the results. Therefore, it is difficult to convince clinical use of PET/MRI and associated techniques without robust evidence. Small population numbers have been reported to lower the ability to detect differences between PD and other similar disorders (thereby not useful for disease differentiation during clinical diagnosis) (Biundo et al. 2021; Garon et al. 2022; Shang et al. 2021b). On the other hand, two other studies found that the small sample size restricted the ability to examine multiple brain regions (therefore potentially missing important ROIs and global changes for disease staging within a clinical setting) (Quarantelli et al. 2022; Rodriguez-Rojas et al. 2020).

Another key challenge is the lack of availability of PET/MRI scanners in centres (particularly in underdeveloped countries) (Mangalore et al. 2022), and the associated requirement for high-end workstations (Wimalarathne et al. 2022). Both the use of PET/MRI systems and high-end workstations for elaborate data processes require expertise. However, with such limitation of availability, it is also difficult to train such skilled technicians.

Although automated and machine-learning algorithms aim to reduce the need for technical or manual work, there are still challenges before it is applicable for widespread use. For example, the trial by Kwon et al. (2016) demonstrated that the use of iso-contouring with max threshold may not fully include the caudate nucleus which may cause significant bias towards underestimating standardised uptake values of PET tracers.

Finally, one inherent challenge not explicitly mentioned in the included studies is that PET/MR imaging seems to be limited to those with early stages of PD. Of all the studies that reported disease stage and symptom severity, only one study included patients with moderate–severe PD (Zang et al. 2022). Without more feasibility studies in more severe cases, it may be difficult to include PET/MRI within a clinical setting for moderate–severe PD patients as these patients may refuse or have difficulties undergoing PET/MRI (e.g. discomfort and therefore perhaps movement during preparation and duration of scan, thereby producing noise or making data unusable). The authors of the Zang study also noted that the 12-h washout period prior to scanning may not be sufficient especially for severe cases. Another study has also raised the concern that the 12-h washout period may not be sufficient and may also be a caveat to accurate results interpretation (Rodriguez-Rojas et al. 2020). This is on the basis that it is widely known that prolonged dopamine treatment can modulate functional networks in PD patients (Rodriguez-Rojas et al. 2020).

### Future directions

There were three articles that proposed future directions towards translational clinical use of PET/MRI systems and techniques in PD. According to An and colleagues, more accurate ultrashort echo time (UTE) segmentation and MR-based attenuation correction can be achieved through further optimisation of the UTE sequences (e.g. by reducing the off-resonance effects, enhancing the robustness of non-Cartesian data acquisition, and fat saturation) (An et al. 2016). Although the method used greatly reduced spatial bias in quantification compared to a previous study by the same study team (Choi et al. 2014), further optimisation can examine smaller structures with minimal partial volume effects and quantification bias (An et al. 2016).

Another way to enhance segmentation performance mentioned by Pan et al. (2022) is to increase the number of atlases using data from a large PD sample. Having more atlases will provide better segmentation accuracy since more PET distribution patterns might be included for PD and its subtypes, and help to distinguish between different parkinsonism disorders (Pan et al. 2022).

Finally, the development and examination of more PET tracers which are specific to non-dopaminergic systems (e.g. serotonergic system, cholinergic system, noradrenergic system) has been suggested in the study by Shang and team (Shang et al. 2021b). This is warranted as these systems are also affected in PD and studying these may demonstrate new underlying mechanisms that may explain the cognitive impairment, mental health conditions, pain, and other sensory dysfunctions of PD in addition to motor function decline.

## Discussion

This study is among the first to examine the specific challenges from primary research studies that has limited the widespread clinical used of PET/MRI for patients with PD. Despite PET/MRI hybrid systems being used since 2006 and a plethora of research boasting its robustness for enhancing clinical practice (particularly in oncology), it is rarely employed for routine PD diagnosis, staging, and progress- and intervention-tracking.

One of the greatest challenges to convince the wide application of PET/MRI is the lack of machine availability compared to other hybrid systems such as PET/CT, and individual MRI and PET scanners. This may be due to expensive machinery, operational costs, and lack of trained expertise (Vitor et al. 2017a; Wimalarathne et al. 2022; Mangalore et al. 2022). It is also questionable as to whether it is even necessary to use PET/MRI, as one study has raised the concern that a limitation to their study was the lack of a healthy control to avoid unnecessary exposure to ionising radiation (Quarantelli et al. 2022). Until more large-scale, robust, and longitudinal studies are conducted using PET/MRI, PET/CT is the more accepted hybrid system to simultaneously acquire metabolic and structural information due to its widespread availability (despite also including exposure to ionising radiation). On the other hand, perhaps the use of PET/MRI hybrid systems are better used for developing automated algorithms and machine-learning tools to be used where PET/MRI scanners are unavailable. For example, the Curved Multiplanar Reconstruction (CMPR) technique can be used where PET is not available (Mangalore et al. 2022). The CMPR technique was shown by Mangalore’s study, to aid early visualisation of subtle patterns of atrophy in numerous dementia conditions which included PD. The use of CMPR does not require sophisticated software and can be done on any workstation, making it useful to communicate with patients in a clinical setting (Mangalore et al. 2022).

Although there are many challenges identified, emerging applications of PET/MRI systems are encouraging greater utility of this powerful tool. A strong advantage of PET/MRI over other imaging techniques, is the TOF reconstruction offered which is particularly useful and crucial when employing short-lived radioligand such as [^11^C]-tracers (Wimalarathne et al. 2022). With the enabling of shorter scan time and simultaneous acquisition of data, it is evidently superior over individual PET and MRI routine scans which can have lengthy preparation, data acquisition, and post-processing times. Of note, however, a PET study noted that methodological sophisticated group analyses and data processing are often not available in most hospital and clinics, thereby making it redundant for individual PD diagnosis in clinical practice (Depierreux et al. 2021). To tackle the arduous workflow from data acquisition to interpretation, the potential solution suggested by Pan et al (2022) shows promise in producing enhanced efficiency and accuracy of PD diagnosis via the use of a fully automated multi-atlas database for PET image analysis (Pan et al. 2022).

With regard to developing new ligands for PD to enable improvements in early and differential diagnosis, there are several studies (in PET trials, but not used in PET/MRI) that are in agreeance with Shang and colleagues. The PET study by Winer’s laboratory in 2018 proposed a tracer that could be useful in PD (Winer et al. 2018). The tracer identified by these authors was [^18^F]AV-1451, which preferentially binds to helical filament tau and associated with alpha-synuclein pathology (rather than all forms of pathological tau which may be due to AD) (Vacchi et al. 2020). Nevertheless, tracers that are more specific to PD such as those for alpha-synuclein pathology are still yet to be developed and examined for human-use (Yagi et al. 2010) as lack of specificity of ligand binding can cause contamination of signal (Schonhaut et al. 2017). On the other hand, the development and examination of more PET tracers which are specific to non-dopaminergic systems (e.g. serotonergic system, cholinergic system, noradrenergic system) has also been suggested (Shang et al. 2021b). This is warranted as these systems may provide new insight into potential mechanisms underlying the cognitive deficits, mental health conditions, pain, and other sensory dysfunctions of PD in addition to motor function decline.

Furthermore, the development and future use of short-lived radio-tracers such as those [^11^-C] variations mentioned above, and the use of faster binding tracers such as [^18^F]LBT-999 (a newly developed alternative to other DAT tracers with 30 min wait-time between injection and scan) are warranted to enhance patient comfort. The uptake and feasibility of [^18^F]LBT-999 has been suggested to enhance clinical utility by offering a desirable shorter time of examinations (Ribeiro et al. 2020). Regardless, whether it is in the development of short-lived radio-tracers or fast-binding ligands, these are invigorating for the future of PD PET/MRI research and clinical practice.

## Conclusion

Taken the combined findings in this review, PET/MRI has shown promising utility for routine clinical application from image reconstruction to the use of fully automated systems, to aid more efficient diagnosis. Nevertheless, there remain challenges, of which many can be resolved through the use of much larger-sampled and longitudinal studies. Meanwhile, future studies are called to develop new fast-binding tracers that have specific affinity to PD pathological processes including those that may help to explain mechanisms underlying clinical manifestations in addition to motor deficits.

## Data Availability

Not applicable.

## Abbreviations

AD: Alzheimer’s disease
[^11^C]-CFT: 11C-2-ß-carbomethoxy-3-b-(4-fluorophenyl) tropane
[^11^C]-PiB: 2-(4-N-[11C] methylaminophenyl)-6-hydroxybenzothiazole
CSF: Cerebrospinal fluid
DTI: Diffusion tensor imaging
DWI: Diffusion weighted imaging
DAT: Dopamine transporter
DAT BR: Binding ratio of dopamine transporter
EPI: Echo planar imaging
ET: Essential tremor
FA: Fractional anisotropy
[^18^F]-DOPA: 3,4‑Dihydroxy‑6‑[^18^F]-fluoro-l‑phenylalanine
[^18^F]-FDG: [^18^F]-Fluorodeoxyglucose
[^18^F]-FE-PE2I: [^18^F]-(E)-N-(3-iodoprop-2-enyl)-2β-carbofluoroethoxy-3β-(40-methyl-phenyl) nortropane
[^18^F]-FP-CIT: [^18^F]-fluoropropyl-carbomethoxyiodophenylnortropane
[^18^F]-FP-DTBZ: [^18^F]-9-fluoropropyl-(+)-dihydrotetrabenazine
[^18^F]-FMM: [^18^F]-flutemetamol
FLD: Frontotemporal lobar degeneration
FLAIR: Fluid attenuated inversion recovery
fMRI: Functional MRI
FOV: Field of view
FSE: Fast spin echo
GE: Gradient echo
HC: Healthy control
LBD: Lewy body dementia
MCI: Mild cognitive impairment
MD: Mean diffusivity
MND: Motor-neuron disease
MPRAGE: Magnetization prepared rapid acquisition gradient echo
MRgFUS: Magnetic resonance-guided focused ultrasound
MSA: Multiple system atrophy
NPH: Normal pressure hydrocephalus
PET/MRI: Positron emission tomography/magnetic resonance imaging
PD: Parkinson’s disease
PSP: Progressive supranuclear palsy
SWI: Susceptibility-weighted imaging
TE: Echo time
TI: Inversion time
TR: Repetition time
UPDRS III: Unified Parkinson’s Disease Rating Scale III for motor examination
UTE: Ultrashort echo time
VMAT2: Vesicular monoamine transporter type 2
